# The application of artificial intelligence in systemic lupus erythematosus: a bibliometric analysis of current trends and future directions

**DOI:** 10.3389/fmed.2026.1929792

**Published:** 2026-08-28

**Authors:** Ziyi Liu, Wenqian Yu, Xinxin Meng, Jiaying Yan, Qiang Fu

**Affiliations:** 1 Dongzhimen Hospital, Beijing University of Chinese Medicine, Beijing, China; 2 Xiyuan Hospital, China Academy of Chinese Medical Sciences, Beijing, China

**Keywords:** artificial intelligence, bibliometrics, precision medicine, systemic lupus erythematosus, visual analytics

## Abstract

**Background:**

Systemic lupus erythematosus (SLE) poses significant clinical challenges due to its heterogeneity and unpredictable relapses. Artificial intelligence (AI) is driving a paradigm shift in SLE management through molecular subtyping and prognostic modeling. However, a comprehensive quantitative analysis of this rapidly growing field is lacking. This study employs bibliometric and visualization methods to systematically map the development, collaboration patterns, and research frontiers of AI in SLE.

**Methods:**

English-language original articles and reviews on AI in SLE, published between January 1, 2005, and June 10, 2026, were retrieved from the Web of Science Core Collection and Scopus. After rigorous screening, 707 core publications were analyzed using R-Bibliometrix, VOSviewer, and CiteSpace to evaluate publication trends, collaboration networks, keyword co-occurrence, and citation bursts.

**Results:**

The 707 included publications demonstrated a polynomial accelerated growth trend since 2005. Research has evolved from algorithmic proof-of-concepts to deep learning and multi-omics integration. China and the United States lead global output and collaboration, with institutions like the Karolinska Institute showing significant impact. Although “machine learning” and “lupus nephritis” remain core topics, the research focus has expanded. Current studies increasingly emphasize molecular stratification, automated pathological image classification, and the longitudinal prediction of flares and organ damage. Recent citation bursts for “immunosuppressive agent,” “tacrolimus,” and “prednisone” highlight personalized treatment efficacy prediction as the latest frontier.

**Conclusion:**

AI has matured from methodological exploration into a robust clinical decision-support tool for SLE, particularly for lupus nephritis assessment and flare prediction. Future advancements rely on conducting prospective clinical validations in real-world cohorts, predicting personalized drug efficacies, and leveraging multi-omics to decode pathological mechanisms. These steps are essential to transition SLE management from traditional empirical approaches to data-driven precision medicine.

## 1 Introduction

Systemic lupus erythematosus (SLE) is a chronic inflammatory disease characterized by multi-organ involvement and autoimmune abnormalities. Its pathogenesis is complex and involves B-cell hyperactivation, T-cell subset dysregulation, immune complex deposition, and the release of inflammatory cytokines (1, 2). Current epidemiological data indicate that the global incidence of SLE is approximately 5.14 per 100,000 person-years, with an overall prevalence of approximately 43.7 per 100,000. It is estimated that there are currently approximately 3.4–5 million people worldwide with various forms of lupus, with SLE accounting for about 70% of all cases. SLE can lead to multisystem involvement; clinical epidemiological studies have observed that approximately 50% of SLE patients will experience damage to major target organs—such as the heart, lungs, kidneys, or central nervous system—during the course of the disease (3, 4). Additionally, musculoskeletal manifestations, particularly joint involvement, are exceptionally common and can affect up to 90% of patients (5). Currently, there is no cure for this condition. While the use of glucocorticoids and traditional immunosuppressants can significantly improve patients' 5-year survival rates, long-term use may lead to adverse reactions such as infections, metabolic disorders, and organ toxicity. Furthermore, some patients may even experience disease recurrence or develop refractory forms of the disease (6).

SLE is characterized by complex clinical heterogeneity and unpredictable relapses. Consequently, traditional statistical models and experience-based diagnostic and treatment approaches are insufficient for precise clinical management (7). In recent years, the application of Artificial Intelligence (AI) technologies—particularly Machine Learning and Deep Learning—has provided new methodological support for addressing this clinical challenge (8, 9). AI algorithms are capable of processing high-dimensional data and recognizing nonlinear patterns, enabling them to effectively integrate clinical and biological big data sources such as electronic health records, medical images, and multi-omics data. Currently, AI is widely used in the research and clinical management of SLE (10). First, with regard to disease phenotypes and diagnosis, AI helps stratify SLE patients into molecular subtypes by analyzing large-scale clinical and molecular data, thereby aiding in early diagnosis and identifying potential therapeutic biomarkers (11). Second, in terms of assessing target organ involvement, image analysis technologies based on computer vision and deep learning have been applied to the automated identification and histopathological grading of lupus nephritis (LN) tissue sections. This automation improves the objectivity and consistency of clinical histological diagnoses (12). In addition, AI predictive models built using longitudinal follow-up data can be used to effectively assess patients' cumulative risk of long-term organ damage and the probability of disease recurrence (13, 14). The application of these technologies has, to a certain extent, improved the accuracy of assessing the complex nature of SLE.By providing decision-making support to clinicians in developing personalized treatment plans, these tools promote the further advancement and development of precision medicine for SLE.

Although the potential applications of AI in SLE are widely recognized, there is currently a lack of systematic integration of the massive volume of literature that has emerged in the short term. Bibliometric and visual analysis, which combines statistical and network science methods, enables both qualitative and quantitative analysis of large-scale literature data. This approach can illustrate the evolutionary trajectory and collaborative network structures within specific research fields and identify cutting-edge academic trends through relevant algorithms (15). To ensure the reliability and comprehensiveness of the research data, this study conducted a joint search of the Web of Science Core Collection (WoSCC) and the Scopus database. Given the differences in journal coverage and indexing rules among various databases, the research team cross-referenced the search results from these two major sources and performed data cleaning to remove duplicates. This strategy aimed to leverage the complementary nature of the data sources, reduce the risk of literature omission and selection bias associated with relying on a single database, and ensure the completeness of the sample included in the analysis (16). Based on the standardized dataset described above, this study employed tools such as the R-bibliometrix package, CiteSpace, and VOSviewer to conduct bibliometric and visualization analyses of literature related to “the application of artificial intelligence in systemic lupus erythematosus.” This study quantitatively analyzed publication trends, the distribution of core journals, and patterns of academic collaboration among countries and institutions with high publication rates. Simultaneously, by utilizing keyword co-occurrence clustering and co-citation analysis, it systematically mapped the knowledge structure and evolutionary patterns of this interdisciplinary fields. This study aims to provide an academic reference for rheumatologists and researchers in related interdisciplinary fields, ultimately offering decision support for the clinical translation of AI-based diagnostic and therapeutic technologies in precision medicine for systemic lupus erythematosus.

## 2 Materials and methods

### 2.1 Data sources and search strategy

This study used the WoSCC and Scopus databases as its primary data sources. The search strategy combined subject headings with free-text terms, focusing on the two core areas of “systemic lupus erythematosus” and “artificial intelligence.” In WoSCC, the search query was set as TS = ((“systemic lupus erythematosus” OR “SLE” OR “lupus erythematosus disseminatus” OR “lupus nephritis” OR “neuropsychiatric systemic lupus erythematosus” OR “NPSLE”) AND (“artificial intelligence” OR “AI” OR “machine learning” OR “Computer Reasoning” OR “Machine Intelligence” OR “Computational Intelligence” OR “deep learning” OR “neural network*” OR “convolutional neural network*” OR “random forest” OR “support vector machine” OR “natural language processing” OR “computer vision” OR “predictive model*”)). In Scopus, the search query is set to TITLE-ABS-KEY(“systemic lupus erythematosus” OR “SLE” OR “lupus nephritis” OR “systemic lupus” OR “disseminated lupus erythematosus”) AND TITLE-ABS-KEY(“artificial intelligence” OR “AI” OR “machine learning” OR “Computer Reasoning” OR “Machine Intelligence” OR “Computational Intelligence” OR “deep learning” OR “neural network*” OR “convolutional neural network*” OR “random forest” OR “support vector machine” OR “natural language processing” OR “computer vision” OR “predictive model*”) AND (DOCTYPE(ar) OR DOCTYPE(re)) AND PUBYEAR > 2004. Searches were conducted using the title, abstract, and keyword fields. The search period was uniformly set from January 1, 2005, to June 10, 2026, and only literature published in English was included.

### 2.2 Literature screening and data cleaning

The literature search for this study spanned from January 1, 2005, to June 10, 2026. Strict inclusion criteria were established for the Web of Science Citation Categories (WoSCC) and Scopus databases: (1) Focus on the application of artificial intelligence in SLE; (2) Literature types were limited to peer-reviewed original research articles and reviews; (3) Published in English. The initial search yielded a total of 1,011 records in WoSCC and 1,277 records in Scopus. After excluding articles that did not meet the type and language requirements and screening titles and abstracts, 512 and 594 records were retained from the two databases, respectively. To improve transparency and reproducibility, a combined automated and manual data-cleaning workflow was implemented. The specific steps were as follows: (1) Data Integration: Raw data files exported from WoSCC and Scopus utilize different field tags. We mapped their corresponding fields (such as title, author, abstract, publication year, and DOI) to a unified set of column names, allowing the two datasets to be concatenated into a single data frame. (2) Automated Deduplication: Python (version 3.11) was used for the initial screening. Records were first deduplicated based on DOIs. For articles lacking DOIs, duplicates were identified through exact string matching of the lowercase article title and publication year. Incomplete records (e.g., missing authors or publication years) and retracted articles were automatically excluded. (3) Manual Review: Two researchers (Z.Y. L. and W.Q.Y.) independently checked the dataset to remove any residual duplicates caused by minor spelling differences and to correct formatting inconsistencies. Disagreements were resolved through discussion with a third researcher (Q.F.) to reach a consensus.

### 2.3 Analysis tools and methods

In this study, the 707 valid articles were imported into several tools, primarily including R-Bibliometrix (Version 5.0), CiteSpace (Version 6.3.R3), VOSviewer (Version 1.6.20), Pajek (Version 6.01), and Scimago Graphica (Version 1.0.53). R-Bibliometrix was primarily used for metadata extraction and basic descriptive statistics, and to identify core academic journals in the field based on Bradford's Law. CiteSpace analyzed the most prominent authors, keywords, and references; visualized keyword networks; generated timelines and temporal zone diagrams; and performed cluster analysis of references. VOSviewer is responsible for constructing and displaying collaboration networks among countries, institutions, and authors, as well as co-occurrence networks for core keywords. Pajek serves as a supplementary tool to adjust the layout of the generated visual network matrices and optimize their clarity. Scimago Graphica integrates geospatial data to visually present the publication volumes of different countries and the distribution of international collaborations. Throughout the analysis, specific thresholds (e.g., minimum publication counts or keyword co-occurrence frequencies) were implemented during network construction. The primary justification for these cut-off values was to filter out peripheral nodes with low statistical significance, thereby minimizing network sparsity and noise. The specific thresholds were systematically determined to balance network modularity with visual readability, ensuring that the retained core components effectively represent the most robust collaborative partnerships and dominant research themes in the field without compromising data integrity.

## 3 Results

### 3.1 Overview of the bibliometric results

**Figure 1:**
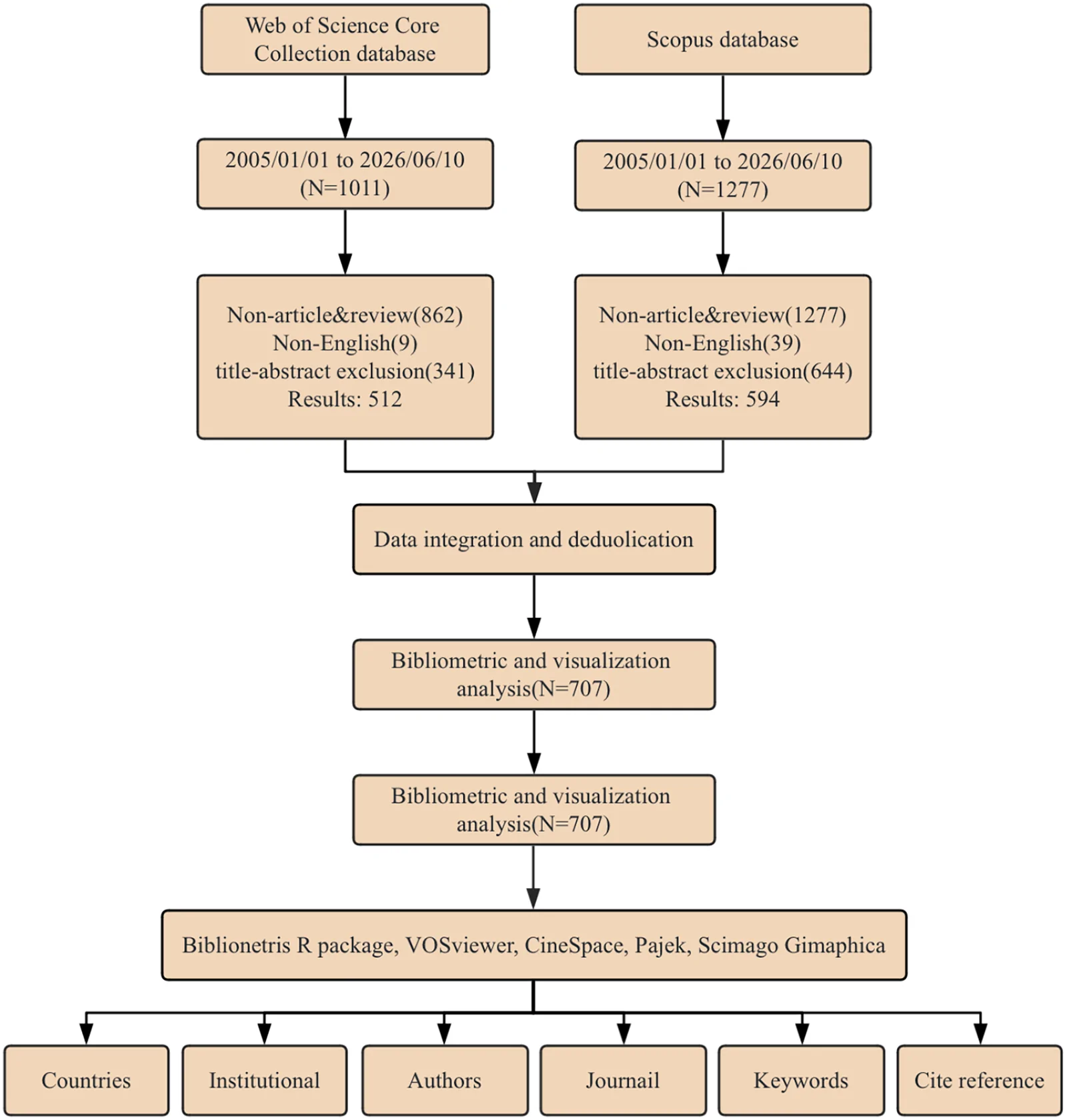
Flowchart of data collection and study design.

This study included a total of 707 publications related to the application of AI in the field of SLE between January 1, 2005, and June 10, 2026. The overall data collection and screening process is illustrated in Figure 1. Of these, 658 (93.1%) were original research articles, and 49 (6.9%) were review articles. Basic information is presented in Table 1.

**Table 1:** General characteristics of the analyzed publications.

Description	Results
Main information about data	
Timespan	2005-2026
Sources (Journals, Books, etc)	298
Documents	707
Annual Growth Rate %	20.5
Document Average Age	3.1
Average citations per doc	13.8
References	30,226
Document contents	
Keywords Plus (ID)	3,699
Author’s Keywords (DE)	1,536
Authors	4,171
Authors of single-authored docs	4
Authors collaboration	
Single-authored docs	5
Co-Authors per Doc	8.5
International co-authorships %	13.7
Document types	
Article	658
Review	49

ID, keywords plus; DE, author’s keywords; Docs, documents.

**Figure 2:**
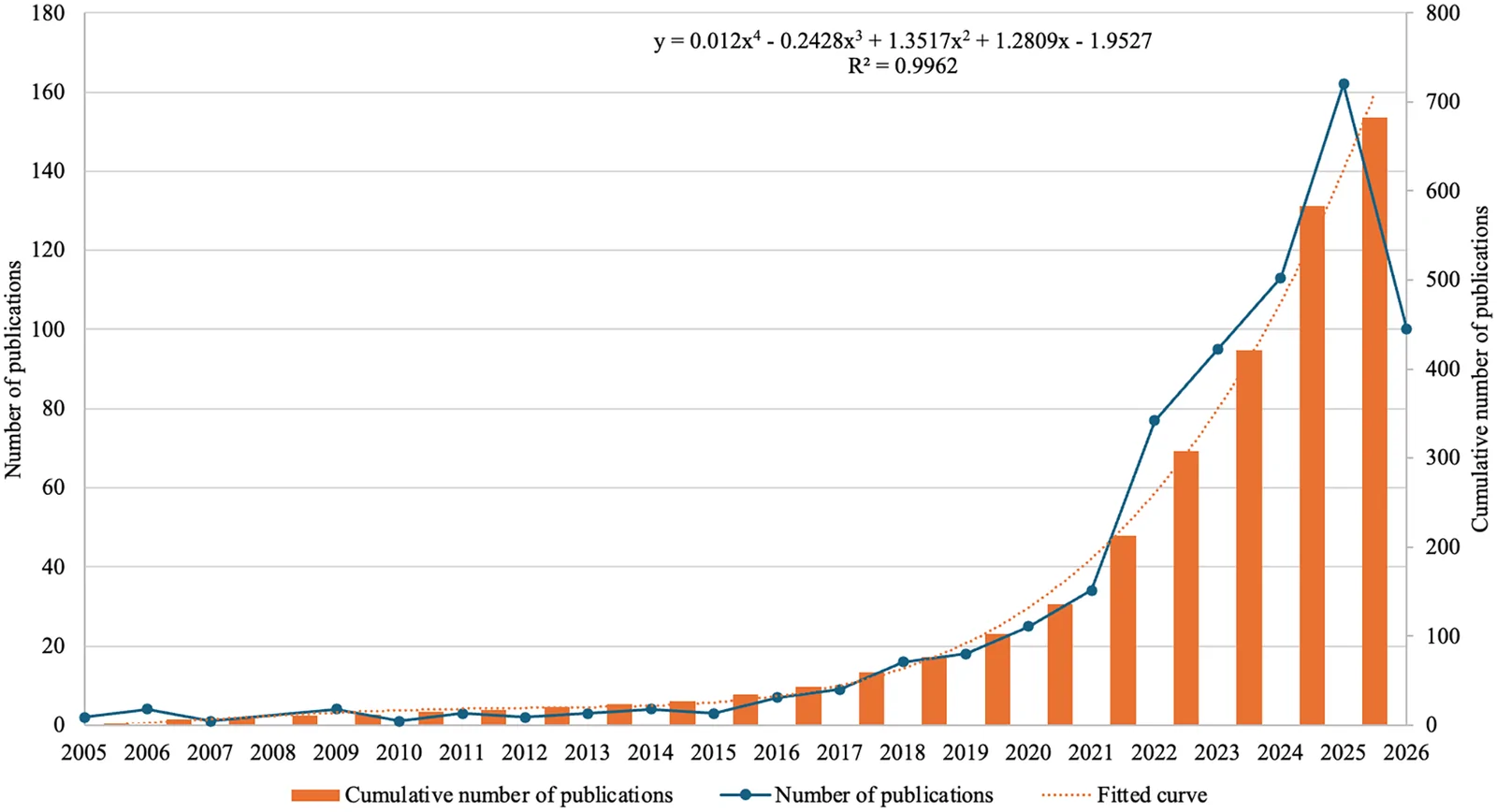
Annual and cumulative numbers of publications concerning artificial intelligence in systemic lupus erythematosus (2005–2026) with a polynomial fitting curve.

As shown in Figure 2, both the annual output and cumulative total of publications exhibited a sustained upward trend. The evolution of this field can be objectively divided into three main periods. The first phase was the initial exploration phase (2005–2016), during which the growth in literature output was relatively gradual, and the annual publication volume remained at a low level for an extended period. This reflected that the application of AI algorithms in clinical and basic research on SLE had not yet reached a significant scale at that time, with efforts primarily focused on proof-of-concept studies and preliminary methodological attempts. The second phase was the steady takeoff phase (2017–2020). This stable upward trend indicates that the academic community's recognition of the potential of AI technology in data mining for autoimmune diseases was gradually deepening. The third phase is the accelerated expansion phase (2021–present). The steep upward trajectory highlights rapid field development, with the apparent decline in publications in 2026 solely reflecting the mid-year data retrieval cutoff (June 10, 2026).

To quantitatively assess the developmental dynamics and future trends in this research field, this study conducted a regression fitting analysis on historical data using Microsoft Excel 2021 (Microsoft Corporation, Redmond, WA, USA). To further quantify the growth dynamics, the Relative Growth Rate (RGR) and Doubling Time (Dt) were calculated across the distinct developmental phases. The RGR increased steadily from 0.26 in the initial phase (2005–2016) to 0.36 in the recent accelerated expansion phase (2021–2025). Concurrently, the Dt decreased from 2.7 years to 1.9 years. These metrics mathematically confirm the field's rapid acceleration, and the decreasing time required for the literature base to double. Finally, the best-fit model for this dataset is a polynomial curve (R² = 0.9962). This high degree of fit mathematically confirms that academic output in this field over the past two decades has followed a polynomial accelerated growth pattern, suggesting the development potential of interdisciplinary research on AI-assisted SLE management.

### 3.2 Bibliometric analysis of countries and regions

**Figure 3:**
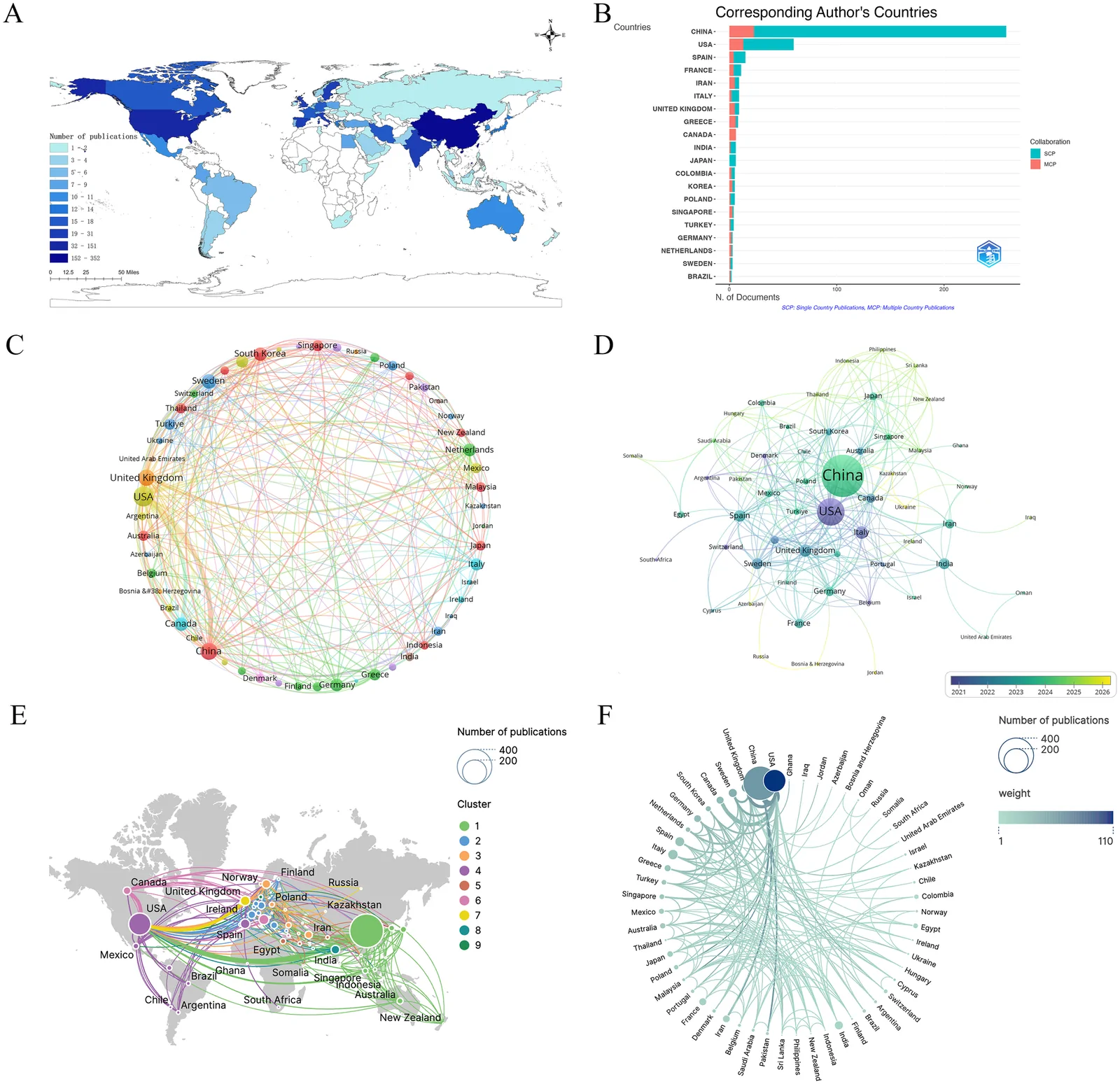
Analysis of AI research applications in SLE by country/region. **(A)** Geographic distribution of publications by country/region worldwide. **(B)** Top 20 countries by number of corresponding author publications and distribution of collaboration patterns. **(C)** Collaboration network map by country/region. **(D)** Distribution of emerging countries/regions. **(E)** Global research collaboration network layout based on geospatial clustering. **(F)** Map of global academic collaboration intensity and network topology.

The global geographic distribution map generated using ArcGIS software illustrates the scale of literature output by various countries in the field of AI applications to SLE research (Figure 3A). Based on the geographic distribution of publications, China and the United States constitute the core research forces in the field's global first tier. Combined with data on corresponding author nationality extracted from R-Bibliometrix (Figure 3B). Furthermore, an assessment of single-country and multi-country publications (SCP and MCP) demonstrates that China and the United States possess robust independent research capabilities while simultaneously serving as the primary drivers of international multi-center collaboration.

The international academic collaboration network reveals that China and the United States occupy central hub positions and maintain the highest level of bilateral collaboration intensity (Figures 3C,F). Additionally, global collaboration exhibits a distinct geopolitical clustering effect (Figure 3E). The United States anchors a Pan-American cluster with extensive connections to Canada and Latin American nations, while China drives an Asia-Pacific cluster characterized by close academic interactions with Australia, India, and Southeast Asia. Meanwhile, European nations form dense, high-frequency sub-clusters within their region. This distribution indicates that geographic proximity significantly shapes transnational academic networks.

Furthermore, temporal tracking of network participation highlights emerging global trends (Figure 3D). In recent years, countries such as Kazakhstan, Azerbaijan, Saudi Arabia, the Philippines, Oman, and Ghana have emerged as new research forces and have become significantly active in this interdisciplinary field. This expansion of the research landscape has objectively benefited from the widespread adoption of open-source AI algorithms globally and the declining costs of cloud computing, enabling more developing countries to leverage their own domestic clinical cohorts of autoimmune diseases to enter the forefront of intelligent, AI-assisted diagnosis and treatment research for SLE.

### 3.3 Bibliometric analysis of institutions

**Figure 4:**
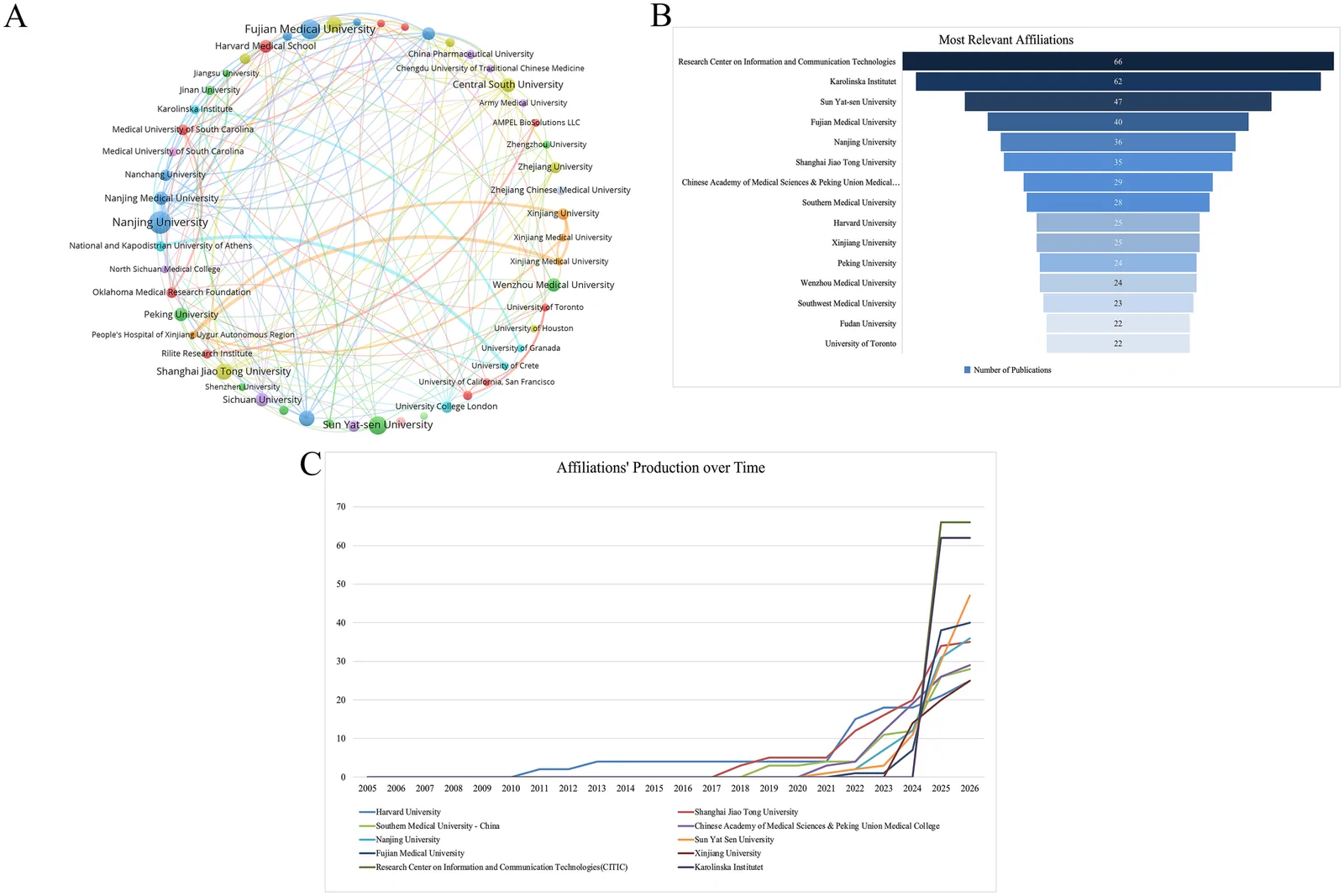
A country/region analysis of AI research in SLE applications. **(A)** Network diagram of institutional collaborations. **(B)** Funnel chart of the top 15 institutions by total publications. **(C)** Line chart of institutional paper output.

Statistical results show that a total of 1,645 institutions participated in research in this field. To filter out peripheral nodes and highlight robust research partnerships, this study set the publication threshold at 5 papers, ultimately identifying 54 core institutions. Based on this, an institutional collaboration network diagram was constructed (Figure 4A); in the network topology, node size represents publication frequency, and line thickness reflects the intensity of collaboration. Observing the clustering patterns, the network exhibits significant geographical clustering. Research institutions within China (such as Peking University, Sichuan University, and Central South University) have formed extremely tightly interwoven secondary collaboration subgroups, which constitute the main framework of the network. At the international collaboration level, some universities have established bilateral or multilateral connections with overseas institutions such as Harvard Medical School and the Karolinska Institute. Notably, the strongest collaborative relationships exist domestically within China; the academic connection between Xinjiang University and Xinjiang Medical University is the closest, followed closely by the partnership between Nanjing University and Nanjing Medical University.

Based on each institution's contributions in this field, we have listed the top 15 most productive institutions by number of publications (Figure 4B). The Research Center on Information and Communication Technologies ranks first in total publications, with 66 papers; Karolinska Institute follows closely behind with 62 publications. Sun Yat-sen University (*N* = 47), Fujian Medical University (*N* = 40), and Nanjing University (*N* = 36) ranked third through fifth, respectively. The data indicate that among the top 15 core institutions, 12 leading institutions are from China, objectively reflecting the outstanding publication productivity of Chinese research teams in this interdisciplinary field; At the same time, Harvard University (*N* = 25) and the University of Toronto (*N* = 22) have also provided significant academic support for the development of this field.

To further explore the entry points and temporal evolution of research capabilities, this study tracked the annual output trajectories of the aforementioned core institutions (Figure 4C). Looking at the development trajectory, Harvard University took the lead in publishing findings in this interdisciplinary field as early as around 2011, demonstrating strong foresight. From 2018 to 2021, Chinese institutions, represented by Shanghai Jiao Tong University and Nanjing University, successively entered a period of heightened activity, driving a steady increase in the volume of publications. It is worth noting that from the end of 2024 through the end of this statistical period, institutions such as the Research Center on Information and Communication Technologies and Karolinska Institute experienced an explosive surge in research in this field, accumulating an exceptionally high volume of output in a short period of time.

### 3.4 Bibliometric analysis of authors

Preliminary statistics indicate that a total of 4,171 scholars have participated in research on artificial intelligence in the field of SLE. In terms of publication frequency, 3,541 authors published only one related paper, accounting for 84.9% of the total number of authors; 630 authors published two or more papers, accounting for 15.1%. According to Price's Law, the minimum number of papers published by a core author in this field is approximately three, identifying a total of 226 core authors. Table 2 presents a quantitative analysis of the top 10 core contributors by combining publication and citation metrics. Geographically, high-output authors in this field are highly concentrated in China, with the notable exceptions of LIPSKY PE and GRAMMER AC from the United States. Although CHEN C from China tops the list with 21 publications, LIPSKY PE achieves the highest fractionalized contribution score (3.18) despite publishing fewer papers. In terms of academic influence, LIPSKY PE ranked first in both global total citations (569) and local citations (51), closely followed by GRAMMER AC with 538 total citations and 48 local citations. As local citation frequency is a key indicator of core influence within a specific subfield, these metrics confirm that LIPSKY PE and GRAMMER AC wield profound academic influence in the interdisciplinary research field of artificial intelligence and SLE and are key scholars driving the development of this field.

**Table 2:** Top 10 authors by publication output in AI in SLE research.

Author	Articles	Articles Fractionalized	Total Citations	Local Citations	Country
CHEN C	21	2.57	148	14	China
LI Y	15	2.25	266	2	China
LIPSKY PE	15	3.18	569	51	USA
CHEN Y	13	1.56	123	1	China
GRAMMER AC	13	2.68	538	48	USA
CHEN H	12	1.40	323	18	China
WANG Y	12	1.72	77	4	China
ZHANG J	12	1.11	113	7	China
ZHANG X	12	1.50	158	9	China
ZHANG Y	12	1.69	43	8	China
CHEN C	21	2.57	148	14	China

**Figure 5:**
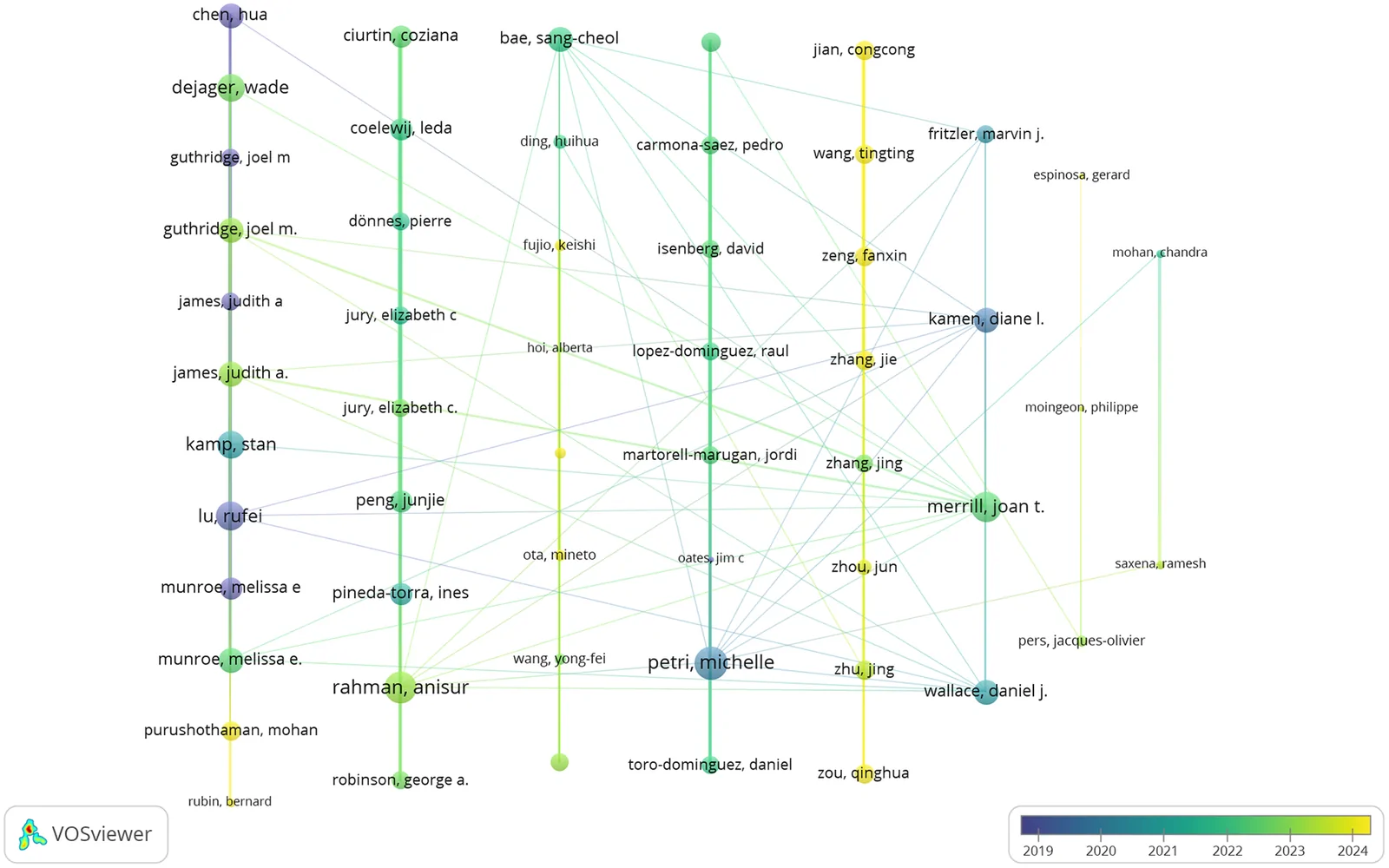
An overlay view of the collaborative networks and timelines of core authors in AI applications in the field of SLE.

To explore the mechanisms of academic collaboration among core research groups, this study mapped the authors' collaboration network and introduced a timeline overlay view to identify active scholars across different time periods (Figure 5). The network topology reveals that nodes centered around Petri, Michelle; Rahman, Anisur; and Merrill, Joan T. are larger in size and densely connected, forming the most critical hubs of the collaborative research network in this field at the present stage. Furthermore, based on the color distribution patterns reflecting temporal evolution (yellow nodes), scholars such as Wang, Tingting; Zeng, Fanxin; Zhang, Jie; and Zhou, Jun form a research group with relatively frequent collaborative interactions in recent years, representing the emerging research forces that have emerged in this field in recent years.

### 3.5 Bibliometric analysis of journals

**Figure 6:**
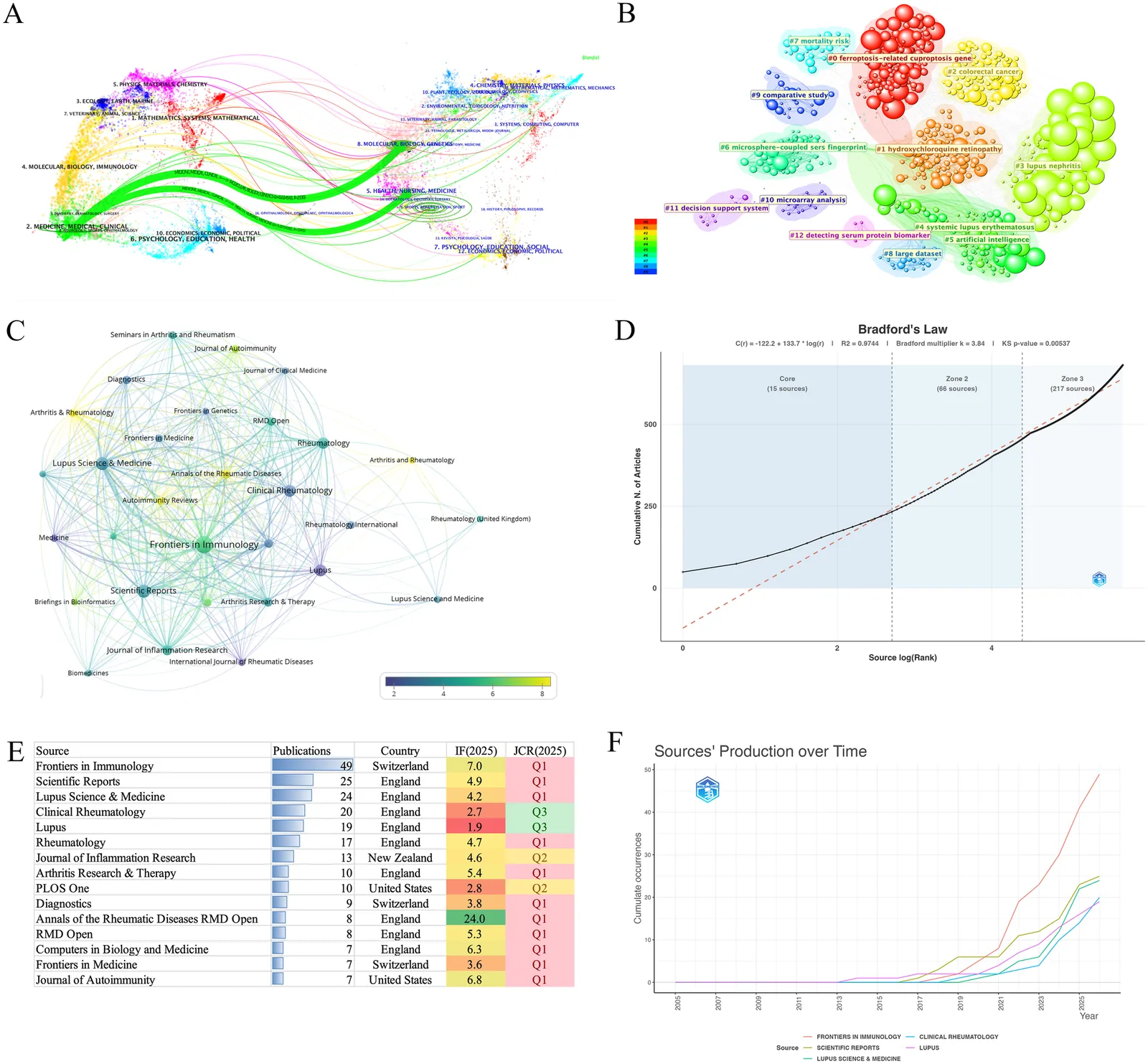
Journal analysis of AI research in SLE applications. **(A)** Dual-map overlay of journals. **(B)** Journal clustering network. **(C)** Overlay map of impact factors based on journal coupling analysis. **(D)** Core journals identified based on Bradford’s Law. **(E)** 15 core journals selected based on Bradford’s Law. **(F)** Source production over time. IF, impact factor; JCR, journal citation reports.

A journal dual-map overlay analysis revealed two core citation pathways in this field (Figure 6A): the knowledge frontiers are primarily concentrated in the “Medicine, Medical, Clinical” and “Molecular, Biology, Immunology” matrices; the corresponding knowledge bases are largely concentrated in the “Molecular, Biology, Genetics” and “Health, Nursing, Medicine” fields. This flow trajectory confirms that this research represents interdisciplinary integration, demonstrating a transition from basic molecular genetics to clinical medicine and immunology. Further in-depth clustering analysis using CiteSpace (*N* = 663, E = 2396) yielded a module value of Q = 0.5978 and an average silhouette value of S = 0.808, indicating a distinct clustering structure and reliable results. The core clusters primarily revolve around themes such as “#0 ferroptosis-related cuproptosis gene,” “#3 lupus nephritis,” “#4 systemic lupus erythematosus,” and “#5 artificial intelligence” (Figure 6B). Additionally, network analysis using VOSviewer revealed that the core citation network consists primarily of high-weight nodes such as Frontiers in Immunology (Figure 6C).

A total of 707 articles were published in 298 journals. Applying Bradford's Law to classify the journal sources revealed that the core zone, second zone, and third zone contained 15, 66, and 217 journals, respectively (Figure 6D). An analysis of the number of articles published in the 15 core journals identified Frontiers in Immunology (49 articles), Scientific Reports (25 articles), and Lupus Science & Medicine (24 articles) ranked in the top three. Furthermore, according to the latest Journal Citation Reports (2025 edition, JCR) released by Clarivate in June 2026, most core journals were ranked in Q1 or Q2, with many based in Switzerland and the United Kingdom. Among these, Annals of the Rheumatic Diseases has the highest impact factor (2025 IF), reaching 24.0 (Figure 6E). In terms of temporal trends, the number of publications in major journals saw significant growth in 2019, with Frontiers in Immunology in particular experiencing an exponential surge after 2021 (Figure 6F).

### 3.6 Bibliometric analysis of keywords

**Figure 7:**
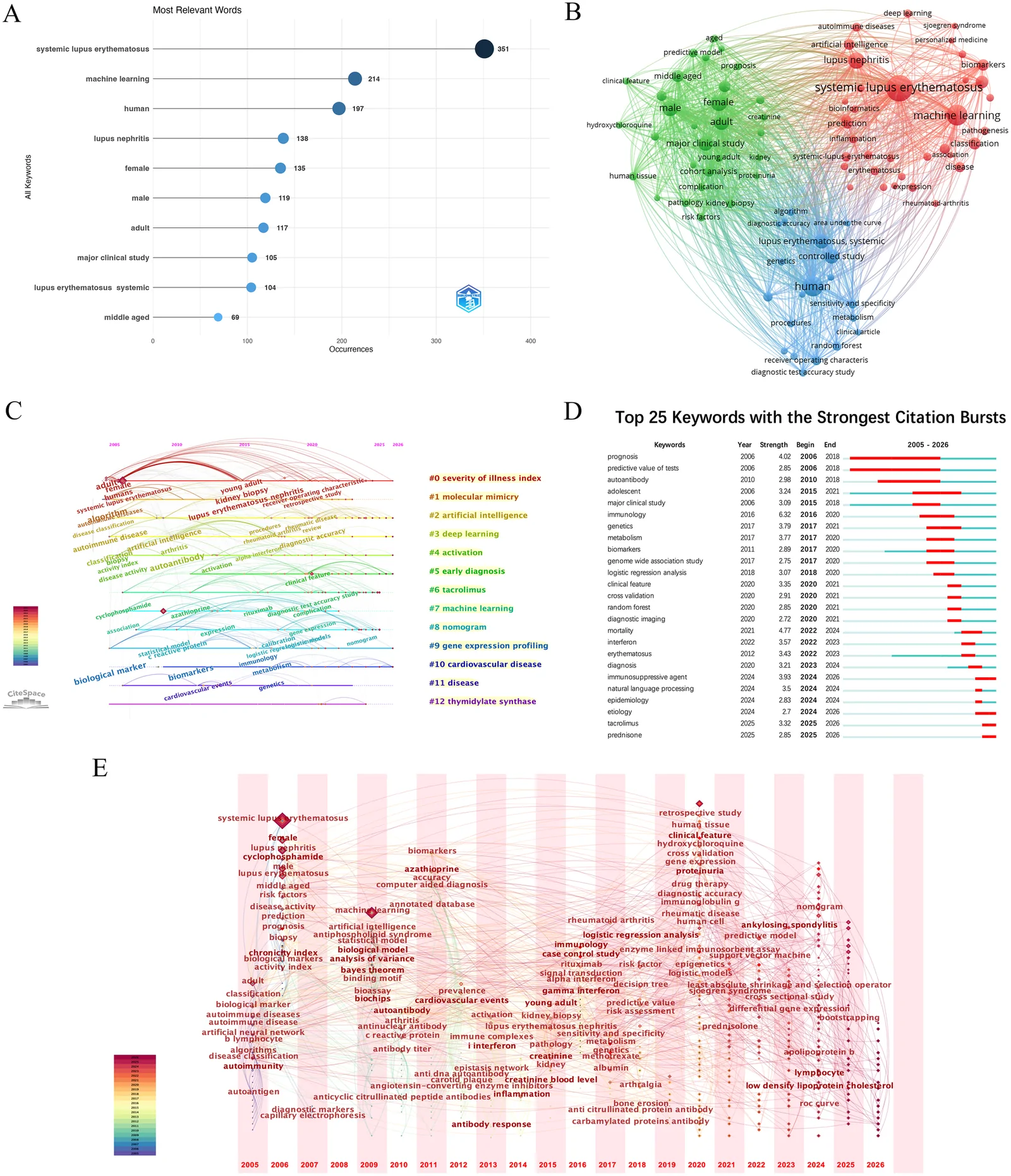
Keyword analysis of AI research in SLE applications. **(A)** Top 10 most frequent keywords in the research field. **(B)** Co-occurrence network and clustering analysis of keywords. **(C)** Evolution of trend topics over time. **(D)** Top 25 keywords with the strongest citation bursts. **(E)** Time-zone view of keyword co-occurrence displayed by year.

Through a co-occurrence analysis of keywords retrieved from 707 documents, a total of 4,823 keywords were identified. To balance network visual readability and capture the dominant research themes, a co-occurrence threshold of 25 was applied, resulting in 70 core keywords. As shown in the high-frequency term statistics in Figure 7A, “systemic lupus erythematosus” (351 occurrences) and “machine learning” (214 occurrences) are the two core nodes, followed closely by “human” (197 occurrences), “lupus nephritis” (138 occurrences), and demographic terms (“female” 135 occurrences, “male” 119 occurrences). Subsequently, the visual network map of these 70 keywords, shown in Figure 7B, reveals three major clusters: the red cluster represents technological and mechanistic frontiers (with dominant terms including “artificial intelligence” and “deep learning”); the green cluster focuses on clinical and pathological characteristics (including “female,” “prognosis,” and “kidney biopsy”); and the blue cluster centers on clinical study design and diagnostic algorithms (including “controlled study,” “random forest,” and “diagnostic accuracy”).

In terms of temporal evolution, the timeline plot (module value Q = 0.6035, average silhouette S = 0.8434) identified a total of 13 significant clusters, primarily covering branches such as “#2 artificial intelligence,” “#3 deep learning,” and “#7 machine learning” (Figure 7C). To further highlight specific trends at the forefront of the field, keyword citation emergence analysis was conducted (Figure 7D). Among the top 25 emerging terms, “immunology” had the highest historical emergence intensity (6.32, active from 2016 to 2020), followed by “mortality” and “prognosis.” Notably, terms that have recently emerged and are projected to continue through 2026 primarily include “immunosuppressive agent” (intensity 3, starting in 2024), “tacrolimus” (intensity 3.32, starting in 2025), “prednisone” (intensity 2.85, starting in 2025), and “etiology” (intensity 2.70, starting in 2024). This trend indicates that, while deeply integrating artificial intelligence technologies, the current cutting edge of the field is shifting back toward the precise application of immunosuppressants and the exploration of disease pathophysiological mechanisms.

Furthermore, combined with the macroscopic evolution trajectories shown in the time-zone plot (Q = 0.939, S = 0.9311) (Figure 7E), an overall paradigm shift can be observed: early research (2005–2007) focused on disease-based phenotypes and traditional drug therapies (such as cyclophosphamide); during the middle phase, concepts such as machine learning and biomarkers were gradually introduced; in recent years (2020 to present), the research focus has clearly shifted toward the construction of clinical predictive models (e.g., nomograms, support vector machines) and the identification of more specific biomarkers (e.g., apolipoprotein B).

### 3.7 Cited references and co-cited references

A citation analysis was conducted on the 707 articles included in this study (Table 3), with “DORIA A, 2006, AM J MED” being the most frequently cited, with 295 citations. Among the metrics assessing recent activity—specifically, the average annual citation frequency (TC per Year)—the data for SOLOMON DH (2020) stood out the most, reaching 26 citations per year. Furthermore, its normalized total citation frequency (NTC) was the highest among the top ten publications (4.23256), indicating that this publication has had a prominent relative influence in recent research. The local citation frequency (LC) reflects a paper's centrality within the specific search dataset under consideration. REDDY BK (2018) has an LC value of 7, while DORIA A (2006) and MUNROE ME (2016) both have LC values of 5, indicating that these papers serve as core pillars of the research topic.

**Table 3:** The top 10 cited publications.

Title	TC	TC per Year	LC	NTC	DOI
DORIA A, 2006, AM J MED	295	14	5	2.94264	10.1016/j.amjmed.2005.11.034
MUNROE ME, 2016, ANN RHEUM DIS	232	21.1	5	2.97982	10.1136/annrheumdis-2015-208140
CARLSEN AL, 2013, ARTHRITIS RHEUM-US	207	14.8	0	1.51463	10.1002/art.37890
VARGHESE SA, 2007, J AM SOC NEPHROL	194	9.7	0	1.00000	10.1681/ASN.2006070767
SOLOMON DH, 2020, NAT REV RHEUMATOL	182	26	0	4.23256	10.1038/s41584-020-0461-x
HUANG KP, 2013, JAMA DERMATOL	180	12.9	0	1.31707	10.1001/jamadermatol.2013.3049
LU R, 2016, J AUTOIMMUN	153	13.9	0	1.96514	10.1016/j.jaut.2016.06.001
LI Y, 2019, CLIN SCI	149	18.6	1	3.60000	10.1042/CS20180841
DEANE KD, 2010, RHEUM DIS CLIN NORTH AM	138	8.1	0	1.00000	10.1016/j.rdc.2010.02.001
REDDY BK, 2018, COMPUT BIOL MED	137	15.2	7	3.94434	10.1016/j.compbiomed.2018.08.029

TC, total citations; LC, local citations; NTC, normalized total citations.

**Figure 8:**
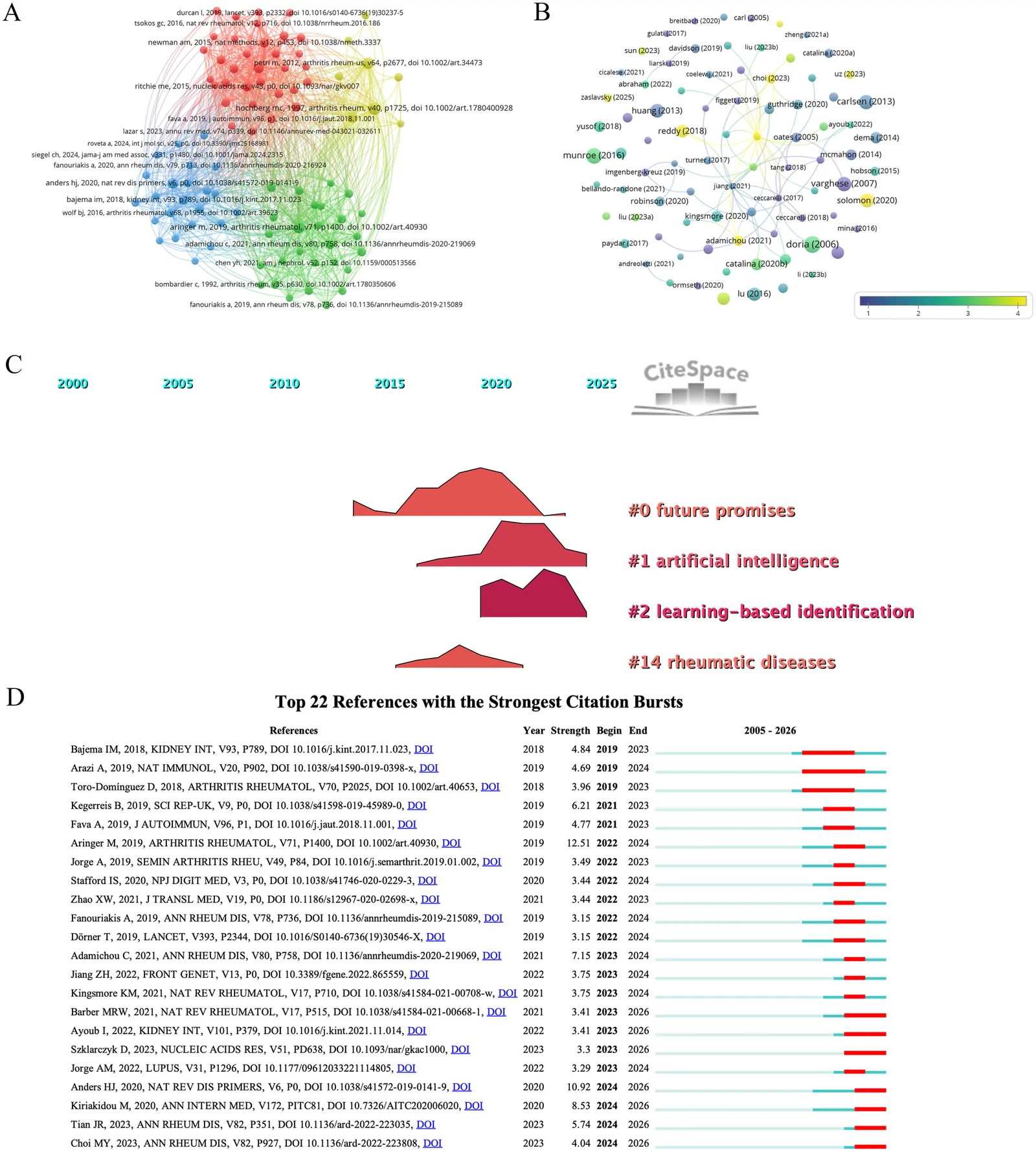
Co-citation analysis of AI research in SLE applications. **(A)** Co-citation clustering plot. **(B)** Plot of normalized citation frequencies. **(C)** Co-citation peak plot. **(D)** Co-citation emergence plot.

The co-cited literature network in Figure 8A reveals four major clusters. Among them, the red cluster focuses on foundational methodologies (e.g., Newman AM, 2015; Petri M, 2012); the green and blue clusters, meanwhile, are densely populated with publications such as Aringer M (2019), Fanouriakis A (2019, 2020), and Anders HJ (2020), indicating that clinical diagnostic criteria and disease management consensus form core subnetworks within the field. In addition, the visualization of the standardized citation frequency distribution in Figure 8B further corroborates the aforementioned findings regarding the high-weight nodes (such as Doria, Reddy, and Munroe), which aligns with the characteristics shown in Table 3. The co-citation peak map (Figure 8C) reveals the trajectory of knowledge evolution between 2000 and 2025. The four main clusters are: “#0 future promises,” “#1 artificial intelligence,” “#2 learning-based identification,” and “#14 rheumatic diseases.” The temporal distribution indicates that the cluster for traditional rheumatic diseases (#14) remains relatively stable, while the peaks of the clusters related to artificial intelligence (#1) and machine learning-based identification (#2) are significantly concentrated between 2018 and 2024, objectively reflecting that the introduction of computational science algorithms has been the core source of knowledge growth and a driving force in this field in recent years.

A citation emergence analysis (Figure 8D) summarized the 22 most prominent emerging publications to identify academic focal points during specific periods. The publication with the highest emergence strength (Strength) was by Aringer M (2019, Arthritis Rheumatol), reaching 12.51 (emergence period: 2022–2024); followed by Anders HJ (2020, strength 10.92) and Kiriakidou M (2020, strength 8.53). In the analysis of current research frontiers, a total of 8 publications are projected to remain prominent through 2026 and are of extremely high reference value. These are Barber MRW (2021), Ayoub I (2022), Szklarczyk D (2023), Jorge AM (2022), Anders HJ (2020), Kiriakidou M (2020), Tian JR (2023), and Choi MY (2023). These consistently highly cited publications objectively reflect the cutting-edge content in the field, indicating that the exploration of disease mechanisms, the validation of algorithmic models, and the latest clinical evidence continue to play a central guiding role in current research.

## Discussion

The deep penetration of AI into the medical field is bringing about a revolutionary technological paradigm shift in the early auxiliary diagnosis of SLE, the precise stratification of molecular subtypes, and the personalized assessment of target organ involvement and recurrence risk. By integrating two authoritative databases (WoSCC and Scopus), this study conducted a comprehensive bibliometric and visualization analysis of 707 core publications spanning from 2005 to 2026. It identified leading countries, high-impact institutions, core journals, and key authors; traced the development trajectory of the field; and clarified research hotspots and future directions.

### 4.1 General information and growth trajectory

Interdisciplinary research on AI in SLE is flourishing, evidenced by a significant and sustained upward trend in related academic publications. This evolution is not only attributable to the ever-expanding global medical big data but is also closely linked to technological breakthroughs in underlying algorithms—such as computer vision and deep learning—as applied to complex autoimmune diseases (17, 18). This study comprehensively reviewed global knowledge output in this field from 2005 to 2026, ultimately including 707 core publications. Quantitative time-series analysis revealed that the annual publication volume in this field follows a highly consistent polynomial accelerated growth model. The high goodness-of-fit mathematically confirms that data-driven technologies are receiving unprecedented attention in SLE management. Furthermore, it objectively indicates that the pace of evolution is continuously accelerating. In terms of its development trajectory, the field has evolved from early-stage proof-of-concept (2005–2016) and the formation of a consensus on its potential (2017–2020) to an exponential growth phase (2021–present). This recent surge is primarily driven by the maturation of deep learning technologies and the integration of multi-omics data. This phased evolution signifies that the application of artificial intelligence in complex clinical scenarios—such as the automated pathological grading of lupus nephritis and predicting recurrence—has become increasingly mature.

### 4.2 Countries and institutions

In the analysis at the national and regional levels, China and the United States constitute the global hubs for AI-assisted SLE research. China ranks first globally in the number of papers with a corresponding author, demonstrating immense capacity for translating research into practical applications; the United States follows closely behind, playing a vital role in laying early theoretical foundations and fostering international collaboration networks. Among the top 15 most productive institutions worldwide, Chinese institutions account for 12 spots, forming the backbone of literature output in this field; institutions such as Harvard University and the University of Toronto provide critical theoretical underpinnings. Furthermore, the Research Center for Information and Communication Technology and the Karolinska Institute have recently experienced explosive growth in publication output, ranking first and second overall. The number of high-level SCPs and MCPs in both China and the United States objectively confirms their capabilities in both independent research and leading global collaboration.

The global scientific research collaboration network exhibits distinct “core-dominated, geo-clustered” characteristics (Figures 3C–E). The underlying reasons for these collaboration patterns are driven by several structural and academic factors. First, the exceptionally strong bilateral connection between China and the United States is largely supported by their substantial research funding allocations and advanced computing infrastructure dedicated to medical artificial intelligence. Additionally, this cross-border collaboration facilitates the integration of complementary expertise, combining advanced algorithmic development with large-scale, diverse clinical cohorts. Second, the densely interconnected European sub-clusters strongly reflect the impact of international collaborative initiatives and shared regional grant programs (such as EU-funded research frameworks), which facilitate cross-border academic partnerships and standardized data sharing. Similarly, geographic proximity and established regional clinical trial networks contribute to the Pan-American cluster centered around the United States. Furthermore, the time-series network (Figure 3D) reveals the recent participation of emerging countries. This expansion can be attributed to the increasing availability of open-source algorithms and the gradual improvement of localized research infrastructure, enabling these nations to utilize their domestic clinical cohorts to validate AI models across diverse ethnic populations. Overall, differences in research funding, infrastructure capacities, regional collaborative policies, and the intrinsic need for interdisciplinary data sharing are key determinants shaping the current global collaboration landscape in this field.

### 4.3 Authors and journals

An analysis of authors and their collaboration networks reveals that there are numerous researchers involved in studies on the application of AI in SLE. While highly productive authors are concentrated in China, U.S. scholars stand out in terms of academic influence. Specifically, CHEN C from China leads globally in terms of output volume with 21 publications. Notably, although LIPSKY PE (15 papers) and GRAMMER AC (13 papers) from the United States do not have the highest total publication counts, they demonstrate exceptionally high academic influence and quantified contributions (3.18 and 2.68, respectively). LIPSKY PE's global total citation count (TC) reached 569, with a local citation count (LC) of 51, ranking first globally; meanwhile, GRAMMER AC follows closely behind with 538 total citations and 48 local citations. This indicates that LIPSKY PE and GRAMMER AC have made outstanding foundational contributions to the interdisciplinary field of AI and SLE. Their work on applying machine learning to analyze the complex heterogeneity of SLE was widely cited shortly after publication, becoming a key cornerstone guiding the development of this field (19, 20). In terms of journal distribution, the 707 retrieved articles were widely distributed across 298 journals, covering multidisciplinary fields such as immunology, rheumatology, clinical medicine, and computational science. Among these, *Frontiers in Immunology* is by far the leading core journal in terms of publication volume in this field, followed closely by *Scientific Reports* and *Lupus Science & Medicine*. This distribution indicates that researchers have a strong preference for publishing findings on AI applications in SLE in high-output, open-access immunology journals and general scientific journals. However, publication volume is not the sole indicator of a journal's academic authority. For example, machine learning-assisted diagnostic models published in *Annals of the Rheumatic Diseases* (2025 IF = 24.0), a top journal in the field of rheumatology, represent the highest level of clinical translation in this field (20).

In addition, journal clustering and dual-graph overlay analysis revealed core thematic clusters such as “ferroptosis-related copper death genes,” “lupus nephritis,” and “artificial intelligence.” The knowledge frontiers are primarily concentrated in the two major matrices of “Medicine/Clinical” and “Molecular/Immunology,” while the knowledge base is largely concentrated in the fields of “Molecular/Genetics” and “Health/Medicine.” This trajectory of knowledge flow clearly corroborates the evolutionary logic of this interdisciplinary research, which is grounded in underlying genetics and molecular biology, deeply integrated with AI technology, and ultimately achieves interdisciplinary translation toward clinical medicine, immunological targets, and the full cycle of disease management (12, 21).

### 4.4 Research themes and emerging trends

Based on the keyword network and high-frequency term statistics, “systemic lupus erythematosus” (351 occurrences) and “machine learning” (214 occurrences) constitute the most central co-occurrence nodes in this field. These terms are closely intertwined with terms such as “lupus nephritis” (138 occurrences) and “female” (135 occurrences), establishing the fundamental research direction in this field: utilizing data-driven models to address the challenges in diagnostic stratification and prognostic assessment of SLE and its core complications, such as lupus nephritis (22, 23). Based on their areas of focus, this fundamental research direction can be broadly divided into two intertwined main strands.

The first strand involves methodological exploration centered on algorithmic evolution and clinical diagnostic support models. Early medical diagnostic support systems largely relied on traditional expert systems or simple linear regression models. However, given the extremely high clinical heterogeneity and complex organ involvement associated with SLE, these models are often limited in their ability to handle high-dimensional, nonlinear data (24). Over time, the keyword time-zone view has clearly outlined the evolutionary trajectory of the underlying clinical algorithms. Beginning in 2009, the emergence of “artificial neural networks” and “support vector machines (SVM)” marked the start of the substantial application of machine learning to data mining in autoimmune diseases. From 2020 onward, the explosive growth of keywords such as “random forest,” “nomograms,” and “predictive models” signaled a profound shift in the field, moving beyond the simple proof-of-concept stage for algorithms toward a comprehensive focus on improving diagnostic accuracy and developing prognostic models in complex clinical settings (4). Currently, multimodal fusion has become a new trend in the development of modern clinical algorithms. This approach simultaneously feeds electronic health records, biochemical test results, and even imaging data from SLE patients into deep learning networks, with the aim of achieving a more precise assessment of target organ damage (25). The second main research track focuses on computational biology explorations centered on multi-omics integration and the investigation of microscopic pathological mechanisms. In SLE research, macro-level clinical signs alone often fail to reveal the underlying causes of disease recurrence and progression. A CiteSpace journal dual graph overlay analysis revealed that a significant portion of the knowledge base in this field originates from the “molecular/genetic” domain. Researchers are no longer content with merely predicting phenotypes; instead, they are turning their attention to deeper-level microscopic pathological mechanisms (26). For example, cutting-edge subfields such as “ferroptosis-related cuproptosis genes” and “apolipoprotein B,” which have been prominently identified through keyword clustering analysis, represent the core areas of current research aimed at screening for novel disease biomarkers by processing massive amounts of transcriptomic and metabolomic data using machine learning (27). This AI-based data mining not only facilitates the molecular subtyping of SLE patients but also provides key insights into the mechanisms underlying the initiation of autoimmune responses and inflammatory damage to target organs at the network level of gene regulation and metabolic pathways.

In summary, the focus of research in this field has shifted from early-stage statistical analysis of single data points—such as basic phenotypes or traditional medications (e.g., cyclophosphamide)—to a comprehensive integration of multimodal methods based on deep learning and ensemble algorithms, with the aim of achieving more in-depth precision screening, mechanism exploration, and dynamic prognosis assessment for SLE. For example, by mining large-scale clinical and molecular features, “diagnostic accuracy” and “sensitivity and specificity” have become central objectives in recent years. AI is widely used to process complex, nonlinear clinical data, assist physicians in early diagnosis, and stratify patients into subtypes based on molecular features, thereby identifying potential therapeutic targets. Meanwhile, regarding complications of “lupus nephritis,” the combination of “pathology kidney biopsy” and deep learning technologies has demonstrated tremendous potential. Computer vision-based image analysis systems have been applied to the automated identification and grading of pathological sections, significantly enhancing the objectivity, consistency, and efficiency of clinical histological diagnosis (28). Furthermore, in day-to-day clinical management, “prognosis” and “mortality” have always been the focus of research (29). Intelligent predictive models built using longitudinal follow-up data effectively assessed the cumulative risk of long-term target organ damage and the probability of unpredictable disease recurrence by extracting time-series features, providing robust decision support for developing personalized follow-up plans.

Of note are current hot topics, including personalized treatment and the evaluation of specific drug efficacy, algorithm diversification, and the monitoring of microscopic pathogenesis and comorbidities. First, among the top 25 emerging terms, with the exception of “immunology” and “mortality,” the terms that have recently emerged and are expected to remain prominent through 2026 are concentrated in the field of drug therapy, such as “immunosuppressive agent,” “tacrolimus,” and “prednisone.” (3, 30, 31) This trend not only reflects clinical needs in addressing the toxic side effects of long-term hormone therapy and traditional immunosuppressants, but also signifies that current AI research—while deeply integrating algorithmic technologies—is shifting its focus from broad-based disease diagnosis back toward precision medication and personalized efficacy prediction. In the future, using algorithmic models to evaluate the clinical efficacy of specific immunosuppressants and glucocorticoids to assist in the development of treatment regimens will remain a key area of research. Second, modeling methods in this field have become increasingly specific in recent years, with key concepts related to predictive modeling and feature selection—such as “predictive model,” “nomogram,” and “least absolute shrinkage and selection operator”—gradually emerging. In addition, “natural language processing” saw a significant surge in citations in 2024. This suggests that future data processing methods will not only rely on conventional numerical medical statistics but will also begin to focus on using natural language processing (NLP) technologies to extract and integrate unstructured clinical data, such as free text. This integration will improve of automatically extracting disease activity assessments and potential risk signals from real-world cohorts (32). Third, interest in exploring “etiology” continues to this day. The CiteSpace timeline view also reveals that research frontiers in recent years have delved into the molecular level, focusing on concepts such as “apolipoprotein B” and “lymphocytes.” Given that deep learning is not only a central node in the co-occurrence network but also a key cluster (#3) spanning the development timeline, computational biology centered on deep learning will be widely applied to multi-omics data and the submolecular level in the future (33, 34). Fourth, regarding long-term disease management, although lupus nephritis remains the central node in research on complications, the CiteSpace clustering results have clearly identified “#10 cardiovascular disease” as a distinct cluster, and the timeline view has recently shown the emergence of the “low-density lipoprotein cholesterol” node (35–37). This suggests that the scope of intelligent assessment in this field is gradually expanding to include the quantitative prediction of long-term systemic comorbidity risks, such as lipid metabolism abnormalities and cardiovascular disease (38, 39). Real-world, evidence-based data indicate that Gradient Boosting Machines (GBM) and Random Forest models—trained using clinical biochemical markers and large-scale cohort follow-up data—have demonstrated high reliability and clinical interpretability in the early, precise prediction of cardiovascular risk in patients with SLE (40, 41).

### 4.5 Outlook on future development trends

Based on the dynamics of keyword emergence intensity (Figure 7D), temporal co-occurrence trends (Figure 7E), and co-citation knowledge networks (Figure 8), the future evolution of artificial intelligence in SLE will primarily follow three data-driven trajectories.

First, transitioning from retrospective modeling to real-world, dynamic clinical validation. As illustrated in the keyword time-zone view (Figure 7E), recent research focus has distinctly shifted toward methodological terms like “predictive model,” “nomogram,” and “prognosis.” While current machine learning models demonstrate strong performance in static risk prediction using retrospective electronic health records, the bibliometric data suggests a necessary evolution. The sustained high citation of clinical consensus and disease management papers identified in Figure 8 (such as Aringer M, 2019; Anders HJ, 2020) underscores the field's strict demand for clinical reliability. Future research must prioritize prospective clinical validations. Integrating longitudinal time-series data and validating these intelligent models in multi-center real-world cohorts will be essential to translate algorithmic predictions from pure computational concepts into regulated, daily rheumatology management tools.

Second, deepening the exploration of disease mechanisms and precision medication. The citation burst analysis (Figure 7D) reveals a fascinating trend: alongside foundational terms, specific pharmacological and mechanistic terms such as “etiology,” “immunosuppressive agent,” “tacrolimus,” and “prednisone” are the most prominent emerging hotspots projected to continue into 2026. This indicates that AI applications are moving beyond basic phenotype classification to address personalized drug efficacy and toxicity prediction. Consistent with the knowledge flow originating from the “Molecular/Genetics” base to the “Medicine/Clinical” frontier in the journal dual-map overlay (Figure 6A), future studies will increasingly utilize machine learning to process multi-omics data. This approach will be crucial for deciphering the underlying microscopic mechanisms of SLE and optimizing individualized dosages for targeted therapies and immunosuppressants.

Third, expanding predictive intelligence to long-term systemic comorbidities. The CiteSpace clustering results clearly identified “#10 cardiovascular disease” as a distinct thematic cluster, and the timeline view recently highlighted nodes like “low density lipoprotein cholesterol” (Figure 7E). This data-driven evidence suggests that the scope of intelligent assessment is gradually expanding beyond acute flare predictions to include the quantitative risk modeling of long-term systemic complications, such as lipid metabolism abnormalities and cardiovascular events. Leveraging AI to monitor and predict these lifelong comorbidities will be a critical frontier in comprehensively improving the life expectancy and quality of life for SLE patients.

### 4.6 Limitations

Although this study conducted a systematic qualitative and quantitative review of the application of artificial intelligence in the field of SLE using bibliometric and visualization analysis methods, it still has the following limitations. First, there is bias related to search language and databases. This study conducted a combined search of two authoritative databases, WoSCC and Scopus, to complement data sources and reduce selection bias associated with relying on a single database. However, the inclusion criteria strictly limited the study to peer-reviewed original research and reviews published in English. This criterion may have excluded some high-quality research findings published in non-English languages and based on clinical cohorts of autoimmune diseases specific to certain countries or regions, thereby introducing language and regional biases to some extent.

Second, bibliometric metrics are inherently susceptible to citation bias and self-citation. The tendency of researchers to cite already highly cited papers, along with self-citations by specific authors or institutions, may artificially inflate certain impact metrics and influence the topologies of the collaborative networks.

Finally, there is an inevitable time lag in citation-based indicators, which is particularly relevant given the rapidly evolving nature of AI-related research. The visualization tools used in this study, such as CiteSpace and VOSviewer, rely heavily on cumulative citation frequencies to track research frontiers. Because the search period ended on June 10, 2026, some recently published algorithmic breakthroughs in rheumatology have not yet accumulated sufficient citations to be fully reflected in the current knowledge networks.

## 5 Conclusion

This study employs bibliometrics and visualization techniques to systematically map the evolutionary trajectory and academic landscape of artificial intelligence (AI) applications in systemic lupus erythematosus (SLE) from 2005 to 2026. The results indicate that interdisciplinary AI research in this field exhibits a significant polynomial acceleration trend, maintaining an explosive growth rate in recent literature output. The research focus has shifted from early-stage methodological exploration and algorithmic proof-of-concept to clinically oriented studies that leverage advanced deep learning models to achieve automated grading of lupus nephritis pathology slides, precise stratification of multi-omic molecular subtypes, and the prediction of disease recurrence probability and cumulative damage to target organs based on longitudinal data. In the future, it will be imperative to focus on the potential of AI in exploring complex microscopic pathological mechanisms and to accelerate the real-world translation of predictive models into precision medication and the development of personalized treatment plans. Research findings suggest that continued breakthroughs in these cutting-edge fields will powerfully drive the development of precision-based assessment and management of SLE, thereby effectively improving the long-term clinical prognosis of this complex autoimmune disease.

## Data Availability

The datasets presented in this study can be found in online repositories. The names of the repository/repositories and accession number(s) can be found in the article/Supplementary Material.
